# X-ray dark-field chest radiography: a reader study to evaluate the diagnostic quality of attenuation chest X-rays from a dual-contrast scanning prototype

**DOI:** 10.1007/s00330-023-09477-4

**Published:** 2023-02-18

**Authors:** Margarete Kattau, Konstantin Willer, Wolfgang Noichl, Theresa Urban, Manuela Frank, Fabio De Marco, Rafael Schick, Thomas Koehler, Hanns-Ingo Maack, Bernhard Renger, Martin Renz, Andreas Sauter, Yannik Leonhardt, Alexander Fingerle, Marcus Makowski, Daniela Pfeiffer, Franz Pfeiffer

**Affiliations:** 1 Chair of Biomedical Physics, Munich Institute of Biomedical Engineering & School of Natural Sciences, Technical University of Munich, 85748 Garching, Germany; 2 Department of Diagnostic and Interventional Radiology, School of Medicine, Klinikum Rechts Der Isar, Technical University of Munich, 81675 Munich, Germany; 3 Philips Research, 22335 Hamburg, Germany; 4 Institute for Advanced Study, Technical University of Munich, 85748 Garching, Germany; 5 Philips Medical Systems DMC GmbH, 22335 Hamburg, Germany

**Keywords:** Diagnostic X-ray radiology, Thoracic radiography, Comparative study

## Abstract

**Objectives:**

To compare the visibility of anatomical structures and overall quality of the attenuation images obtained with a dark-field X-ray radiography prototype with those from a commercial radiography system.

**Methods:**

Each of the 65 patients recruited for this study obtained a thorax radiograph at the prototype and a reference radiograph at the commercial system. Five radiologists independently assessed the visibility of anatomical structures, the level of motion artifacts, and the overall image quality of all attenuation images on a five-point scale, with 5 points being the highest rating. The average scores were compared between the two image types. The differences were evaluated using an area under the curve (AUC) based z-test with a significance level of *p* ≤ 0.05. To assess the variability among the images, the distributions of the average scores per image were compared between the systems.

**Results:**

The overall image quality was rated high for both devices, 4.2 for the prototype and 4.6 for the commercial system. The rating scores varied only slightly between both image types, especially for structures relevant to lung assessment, where the images from the commercial system were graded slightly higher. The differences were statistically significant for all criteria except for the bronchial structures, the cardiophrenic recess, and the carina.

**Conclusions:**

The attenuation images acquired with the prototype were assigned a high diagnostic quality despite a lower resolution and the presence of motion artifacts. Thus, the attenuation-based radiographs from the prototype can be used for diagnosis, eliminating the need for an additional conventional radiograph.

**Key Points:**

• *Despite a low tube voltage (70 kVp) and comparably long acquisition time, the attenuation images from the dark-field chest radiography system achieved diagnostic quality for lung assessment.*

• *Commercial chest radiographs obtained a mean rating score regarding their diagnostic quality of 4.6 out of 5, and the grating-based images had a slightly lower mean rating score of 4.2 out of 5.*

• *The difference in rating scores for anatomical structures relevant to lung assessment is below 5%.*

## Introduction

Dark-field radiography is a new imaging modality that is currently being investigated in clinical trials. The underlying image formation is based on ultra-small angle X-ray scattering [1] and is sensitive to structural changes in the lung [2]. It is therefore a promising approach to the early diagnosis of respiratory diseases. The alveolar structure of the healthy lung and associated air-tissue interfaces cause diffuse refraction and produce a distinct dark-field signal. Pathological changes in pulmonary parenchyma are generally accompanied by an alteration of these air-tissue interfaces, affecting the signal accordingly. Studies on several disease models have demonstrated the diagnostic potential of the dark-field modality in small animal and porcine models [2–8]. After a feasibility study, where the first dark-field radiographs of a human lung in a deceased body had been presented [9], the first experimental system for grating-based chest radiography on humans was constructed and commissioned. The potential benefits are evaluated as part of a clinical study investigating the diagnostic value of chronic obstructive pulmonary disease (COPD). It was shown that the technique can detect and quantify emphysema [10, 11] and that the dark-field signal is independent of demographic factors [12].

Due to the inherent properties of grating-based X-ray imaging [1], the prototype is capable of acquiring both a dark-field image and an attenuation image simultaneously. Chest radiography is generally used for the initial assessment of the lung, as the associated radiation dose is comparatively low and the examination procedure is fast and simple. Additionally, it plays a significant role in COPD diagnosis because it is often used to exclude other pathologies with similar symptoms to COPD [13]. Therefore, a high image quality of both modalities is necessary for diagnostic accuracy. The aim of this study was to evaluate the diagnostic quality of the attenuation images. As a reference measure, we used images acquired at a commercial chest radiography system, since they fulfill the current clinical standard in the diagnosis of respiratory diseases.

## Materials and methods

### Dark-field chest radiography prototype

The prototype consists of a conventional radiography setup in combination with a three-grating-interferometer. Due to the presently limited grating size, the prototype uses a slot-scanning technique where the illumination is restricted to the grating area, which is scanned vertically along the patient to achieve the full field of view of 37 × 37 cm^2^ at the patient, producing a sequence of overlapping images. This leads to a local exposure time per pixel of about 1 s and a total acquisition time of about 7 s. More details can be found in [10, 12].

### Patient collective

The clinical study was approved by the institutional review board and the national radiation protection agency. Between October 2018 and April 2019, 65 patients with and without diagnosed COPD were recruited from the radiology and pulmonology departments. All patients gave written informed consent prior to participation.

Of the included patient collective, smaller cohorts have already been reported in previous work, albeit with different primary outcomes as follows: a total of 27 participants were previously studied by [12], who evaluated the characteristics of dark-field chest radiography in healthy humans; a total of 59 participants were studied by [11], evaluating the characteristics of dark-field chest radiography in patients with pulmonary emphysema; a total of 57 participants were also described by [14], who assessed the diagnostic accuracy of dark-field imaging for emphysema diagnosis. The current study evaluates the diagnostic value of the attenuation chest X-rays obtained with the dark-field prototype, which has not been investigated in these previous studies.

### Image acquisition

As part of the clinical study, for each patient, a chest X-ray in posterior-anterior view was obtained consecutively at a commercial radiography system (DigitalDiagnost, *Royal Philips*) and at the clinical prototype. In the commercial system, a tube voltage of 125 kVp was used in accordance with the clinical routine [15]. The nominal pixel size of the flat-panel detector was 148 µm.

To allow a fair comparison between the commercial system and the prototype setup, similar post-processing algorithms (UNIQUE, *Royal Philips*) were applied, as routinely used in commercial chest X-ray systems [16].

The dose for both the attenuation and the darkfield chest radiograph (for the 73 kg reference person) is 35.1 µSv [17]. The corresponding value for a radiograph in the commercial system is 17.6 µSv. We calculated the effective dose for image acquisition at the prototype from the dose area product with a conversion coefficient of 1.5 µSv/(µGy∙m^2^), determined in cooperation with the national radiation protection agency. The corresponding conversion coefficient for the commercial system was measured to be 2.1 µSv/(µGy∙m^2^).

Due to the prolonged total acquisition time of 7 s due to the scanning process, the patients are asked to hold their breath during image acquisition to minimize the probability of motion artifacts. The effectiveness depends on the ability of the patient to hold their breath and can be limited for patients with a more advanced stage of COPD. All patients in this study reported that they felt confident with holding their breath. However, a blurring of the heart contours cannot be avoided, as at least one heartbeat is captured by the local exposure of 1 s.

### Reader study

Five radiologists with 12, 12, 5, 5, and < 1 year of experience independently assessed the attenuation images of both systems. A total number of 130 images were rated. An annotation tool integrated into the clinical database system was used for the readings, with the images shown individually and in random order. It was not indicated with which system the presented image was acquired. The readers were allowed to revise their previous rating in retrospect and stop a reading session at any point. There were no time constraints during the sessions. Based on a previous study by Uffmann et al, a list of the following anatomical structures was created [18]: lung parenchyma, bronchial structures, perihilar vessels, peripheral vessels, costophrenic recess, cardiophrenic recess, retrocardiac area, carina, heart contours, and osseous structures. Their visibility was rated on a five-point scale adapted from the same work:

- 5: excellent visibility
- 4: good visibility
- 3: moderate visibility
- 2: poor visibility
- 1: unacceptable image

Additionally, the overall image quality and the level of motion artifacts, caused by the prolonged image acquisition time of 7 s, were evaluated. In the case of the latter, a score of 5 indicated that the motion artifacts did not decrease the visibility at all with the lower ratings following correspondingly. The readers were also asked to select which system they thought the image was acquired on.

### Statistical analysis

The scales used for the feature grading were considered interval-scaled, in order to obtain refinement between the ratings by calculating the mean overall readers [19]. The statistical difference between the images from the prototype and the commercial system was determined using an area under the curve (AUC) based z-test. The AUC was calculated for each criterion with the Obuchowski method [20]. If there is no statistical difference between the two systems, the AUC values are expected to be close to 0.5. Based on the standard error of the AUC values the *p* values were calculated to determine statistical significance, which was considered if *p* ≤ 0.05.

## Results

### Patient data

The patient demographics are presented in Table 1. We included 65 patients (64 ± 12 years), among which 42 were male and 23 were female. In total, 11 of them were categorized as COPD patients and 51 as patients without COPD. For 3 patients, the available spirometry data was incomplete, prohibiting a classification. The mean age in the collective was 64 years with a standard deviation of 12 years.

**Table 1:** Patient demographics

Total number of participants	65
Men	42
Women	23
Without COPD	51
With COPD	11
Incomplete spirometry data	3
Mean age ± standard deviation in years	64 ± 12

The mean effective dose for the whole patient collective was determined to be (43.8 ± 26.4) µSv for our prototype, resulting in two images of different modalities and (16.4 ± 6.2) µSv for the commercial system. The values are given as mean ± standard deviation.

### Patient images

**Fig. 1:**
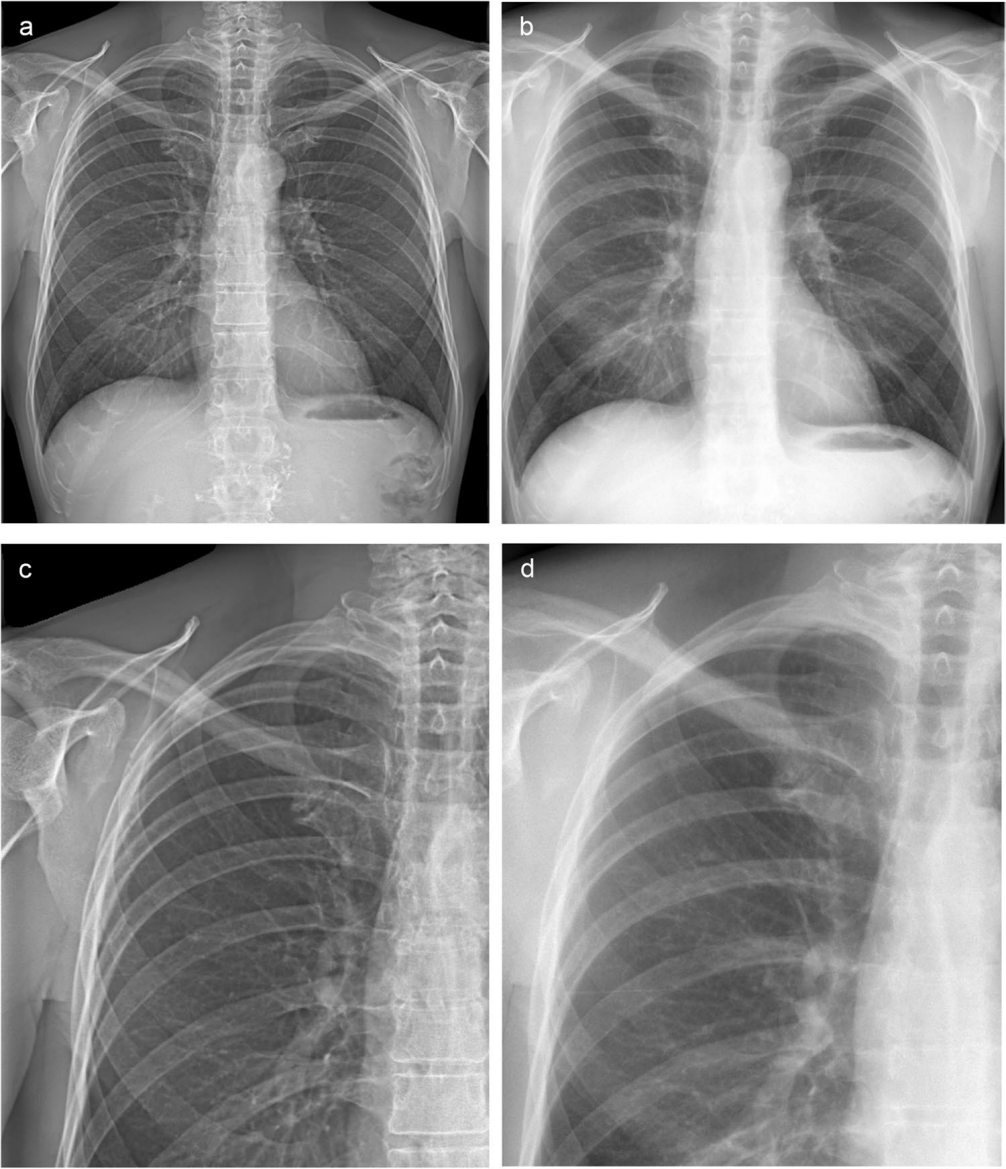
Attenuation images of a male 61-years old patient without lung impairment. **a** Attenuation image acquired with the prototype. **b** Attenuation image acquired at the commercial system. **c** and **d** show enlarged extracts from **a** and **b**, respectively

**Fig. 2:**
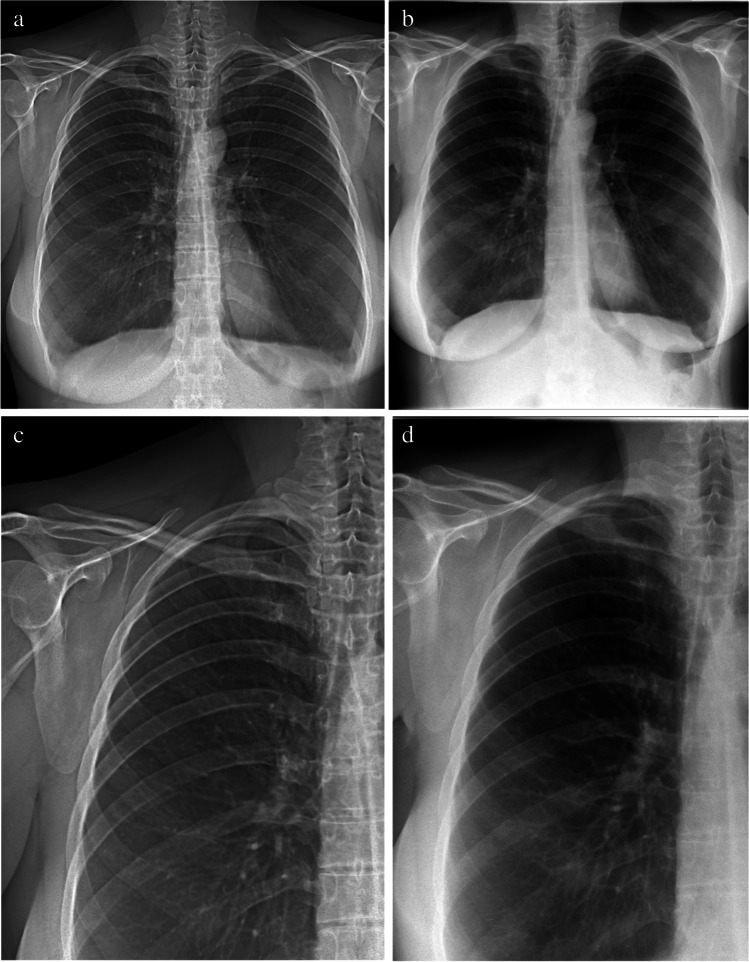
Attenuation images of a female 42-years old patient with COPD (GOLD III). **a** Attenuation image acquired with the prototype. **b** Attenuation image acquired at the commercial system. **c** and **d** show enlarged extracts from **a** and **b**, respectively

Figures 1 and 2 show example images of two patients, one without lung impairment and one with COPD (GOLD III). Visually, a higher contrast can be observed in the images acquired with the grating-based system. Moreover, the lung vessels are not obscured by the ribcage despite the lower tube voltage. Motion artifacts are found as blurring of the heart contours and the diaphragm in the images from the prototype.

### Rating scores

**Fig. 3:**
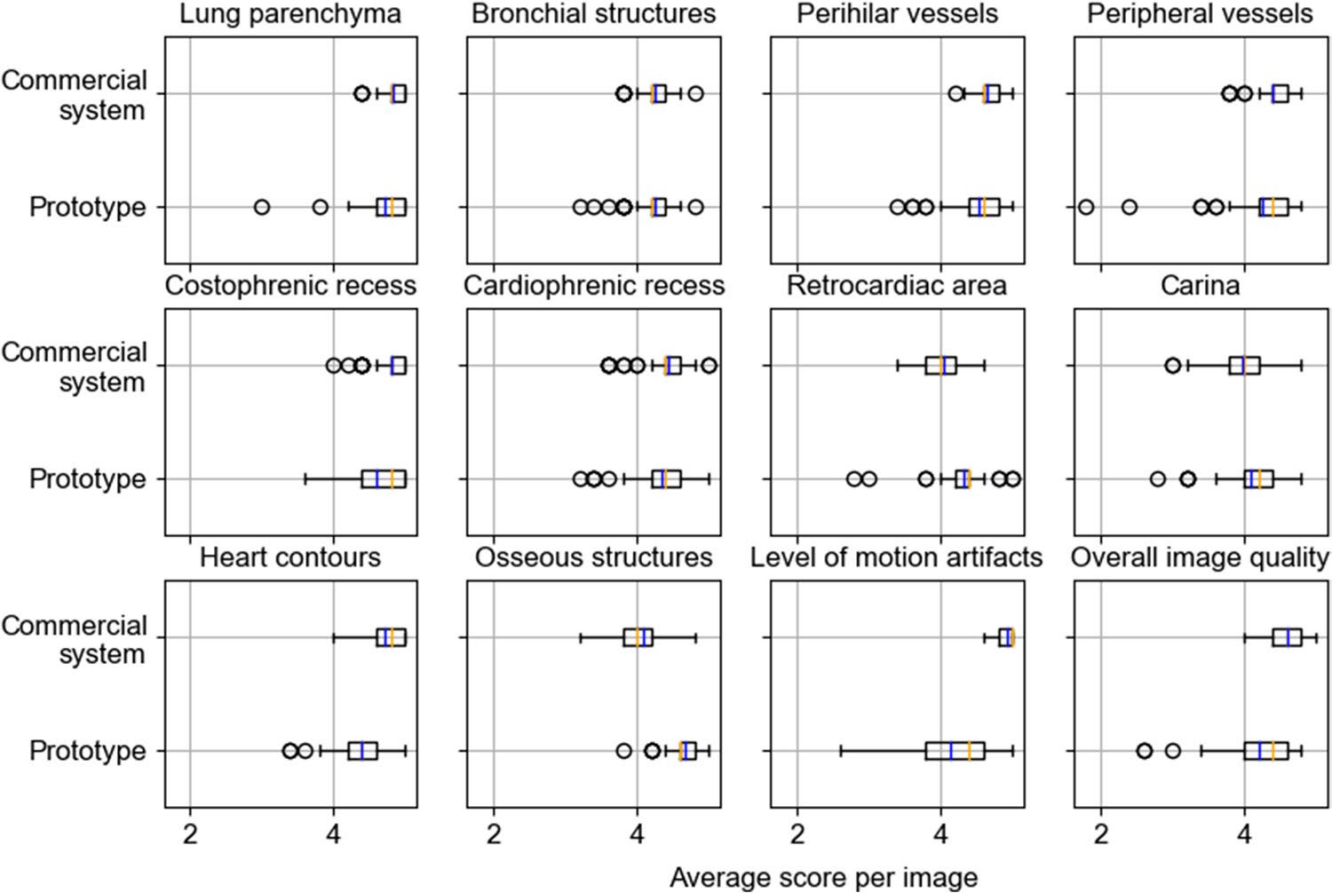
Rating scores per image to visualize image variability averaged over the five readers, compared for every criterion. The circles indicate the outliers of the distribution. The mean and median values are indicated by the blue and orange lines, respectively

The system identification by the readers was correct for 93% of the images. The resulting scores for the anatomical structures averaged over the five readers and their distributions are shown in Fig. 3. The grating-based images obtained a mean score of 4.2 for overall image quality, which is approximately 9% lower than the corresponding mean score of 4.6 for the commercial images. The mean visibility scores were high for both image types, but the ratings were higher for the commercial system for the majority of criteria except for the osseous structures, the carina, and the retrocardiac area. The osseous structures in the grating-based images obtained a mean score of 4.7, which is approximately 13% higher than the corresponding rating of 4.1 for the images of the commercial system. For the majority of criteria, the AUC was close to 0.5 with a *p* value ≤ 0.05, demonstrating that there is no statistical difference between the images from our prototype and the commercial system (Table 2). The ratings for the carina, the cardiophrenic recess, and the bronchial structures resulted in a *p* value > 0.05 with corresponding AUCs in the range between 0.505 and 0.530 and therefore not statistically significant. The AUC for the heart contours, level of motion artifacts, osseous structures, and overall image quality are considerably larger than 0.5.

**Table 2:** Results of the AUC-based z-test evaluating the difference between the attenuation images acquired at the prototype and the commercial system

Rating criterion	AUC	*p* value
Lung parenchyma	0.536	≤ 0.05
Bronchial structures	0.505	0.700
Perihilar vessels	0.532	≤ 0.05
Peripheral vessels	0.542	≤ 0.05
Costophrenic recess	0.573	≤ 0.05
Cardiophrenic recess	0.530	0.052
Retrocardiac area	0.570	≤ 0.05
Carina	0.524	0.080
Heart contours	0.632	≤ 0.05
Osseous structures	0.714	≤ 0.05
Level of motion artifacts	0.778	≤ 0.05
Overall image quality	0.641	≤ 0.05

For the assessment of motion artifacts, the images from the commercial system achieved a mean rating of 4.9, whereas the grating-based images scored 4.1 on average, accounting for a difference of 16%. The spread in visibility score is considerably higher for the grating-based images. However, at the costophrenic recess and the heart contours—areas where motion artifacts are most likely to occur [21]—the difference in image ratings is not as pronounced, both when investigating the spread and the mean values. The grating-based images received mean ratings which were only 4% and 7% lower for the costophrenic recess and the heart contours, respectively.

We were especially interested in the differences between both systems for the costophrenic recess, the bronchial structures, the perihilar, and the peripheral vessels due to their diagnostic relevance in chest radiography [22]. For these anatomical structures, the differences in visibility scores were low (≤ 5%). In general, there is a wider spread in the ratings of the grating-based images with more outliers than in the commercial images. There is a wide spread of the ratings in the level of motion artifacts at the prototype and the corresponding AUC is larger than 0.5. For the osseous structures on the other hand, the prototype consistently receives high ratings, while those for the commercial system vary, also leading to a large AUC.

## Discussion

In this study, we investigated the diagnostic quality of attenuation chest X-ray images acquired with the first clinical dark-field radiography prototype and compared it to corresponding images obtained with a commercial system in a reader study. We found that the visibility of relevant anatomical structures and the overall image quality were rated high for both systems. The presence of motion artifacts in the grating-based images decreased the visibility only slightly.

The improved visibility of the osseous structures in the grating-based images, indicated by a significant difference with a high AUC, is most likely achieved by the lower tube voltage used in the prototype. This confirms the results of Uffmann et al where higher image quality was shown for lower tube voltages [18]. At lower X-ray energies, the photoelectric effect dominates the attenuation cross-section leading to increased absorption and structural contrast. At the same time, Compton scattering is decreased, further improving image quality. The applied image processing enhances the visibility of the lung parenchyma despite the enhanced contrast from the ribcage.

The comparatively high difference in the dispersion of the rating for the peripheral vessels could be due to the lower spatial resolution of the prototype. Additionally, the visibility of these vessels is influenced by involuntary movement due to prolonged image acquisition.

As a result of the short acquisition time in the order of milliseconds in clinical radiography, motion artifacts are not a common problem, which is consistent with the high mean rating score and narrow distribution of ratings for the commercial system in the corresponding criterion. However, they can be an issue in the prototype due to the slot-scanning technique and thus longer acquisition time. Image quality reduction due to these motion artifacts should be reflected in a respectively lower grading in the visibility of the heart contours and the costophrenic recess, which corresponds to the assessment of the diaphragm. Indeed, the commercial images show less variability in the ratings, but the difference between the mean ratings is comparatively small. The variability in the grating-based images is likely caused by single patients that moved during the acquisition. A motion correction algorithm has been developed [23] that automatically detects image locations affected by motion. It can help minimize motion artifacts, reducing the local illumination time to 0.22 s.

Despite the increased presence of motion artifacts and slightly lower average scores in the majority of the criteria, the overall image quality was rated high for the images obtained with our prototype. The difference in image variability for this criterion could be caused by images rated lower due to an increased presence of motion artifacts.

Recent studies on grating-based chest radiography on a deceased human body have shown that the optimal tube voltage for this imaging modality is around 60 to 70 kVp as a compromise between dark-field and attenuation image quality [24, 25]. Moreover, there are several studies indicating that the image quality of attenuation radiographs increases when using lower tube voltages [26–30]. Our reader study results confirm these findings.

The reason for the higher dose in the prototype can be found in its inherent grating configuration, which allows for the extraction of two complementary images instead of one contrast modality only. Nevertheless, the dose is within clinically acceptable ranges [31]*.*

The prolonged acquisition time of our prototype and the need for a 7-s breath-hold poses a significant limitation for clinical practice. Additionally, our study is limited by the small number of patients with COPD. The main aim of this work was to compare the image quality between our prototype and the commercial system. Future studies should investigate the diagnostic value of dark-field radiography for COPD detection which involves a larger population of COPD patients with detailed documentation of their respiratory status.

Overall, our results show that the attenuation images acquired with our prototype are of comparable diagnostic quality to the commercial images. This could potentially simplify the implementation of the system into clinical workflow, as it yields both a dark field and an attenuation image and no additional attenuation X-ray would be required. Moreover, it offers the possibility of perfect registration of both complementary images, providing further potential diagnostic benefits.

## Abbreviations

AUC: Area under the curve
COPD: Chronic obstructive pulmonary disease

## Declarations

### Guarantor

The scientific guarantor of this publication is Franz Pfeiffer.

### Statistics and biometry

No complex statistical methods were necessary for this paper.

### Study subjects or cohorts overlap

Some study subjects or cohorts have been previously reported in Gassert & Urban et al (X-ray Dark-Field Chest Imaging: Qualitative and Quantitative Results in Healthy Humans. Radiology, 2021), Urban & Gassert et al (Qualitative and Quantitative Assessment of Emphysema Using Dark-Field Chest Radiography. Radiology, 2022), Willer & Fingerle et al (X-ray dark-field chest imaging for detection and quantification of emphysema in patients with chronic obstructive pulmonary disease: a diagnostic accuracy study. The Lancet Digital Health, 2021), Urban & Sauter et al (submitted).

### Methodology

• retrospective

• observational

• performed at one institution
